# Recent Advancements in Mitochondria-Targeted Nanoparticle Drug Delivery for Cancer Therapy

**DOI:** 10.3390/nano12050743

**Published:** 2022-02-23

**Authors:** Jiangsheng Xu, James G. Shamul, Elyahb Allie Kwizera, Xiaoming He

**Affiliations:** 1 Fischell Department of Bioengineering, University of Maryland, College Park, MD 20742, USA; jx16@umd.edu (J.X.); jshamul@umd.edu (J.G.S.); akwizera@umd.edu (E.A.K.); 2 Marlene and Stewart Greenebaum Comprehensive Cancer Center, University of Maryland, Baltimore, MD 21201, USA

**Keywords:** mitochondria, nanoparticle, cancer therapy, photothermal, photodynamic

## Abstract

Mitochondria are critical subcellular organelles that produce most of the adenosine triphosphate (ATP) as the energy source for most eukaryotic cells. Moreover, recent findings show that mitochondria are not only the “powerhouse” inside cells, but also excellent targets for inducing cell death via apoptosis that is mitochondria-centered. For several decades, cancer nanotherapeutics have been designed to specifically target mitochondria with several targeting moieties, and cause mitochondrial dysfunction via photodynamic, photothermal, or/and chemo therapies. These strategies have been shown to augment the killing of cancer cells in a tumor while reducing damage to its surrounding healthy tissues. Furthermore, mitochondria-targeting nanotechnologies have been demonstrated to be highly efficacious compared to non-mitochondria-targeting platforms both in vitro and in vivo for cancer therapies. Moreover, mitochondria-targeting nanotechnologies have been intelligently designed and tailored to the hypoxic and slightly acidic tumor microenvironment for improved cancer therapies. Collectively, mitochondria-targeting may be a promising strategy for the engineering of nanoparticles for drug delivery to combat cancer.

## 1. Introduction

Mitochondria are essential subcellular organelles for the life and death of cells [1,2]. Since mitochondria are traditionally considered the “powerhouse” of most eukaryotic cells and are responsible for cellular respiration, they can also mediate apoptosis and have been identified as a valuable target for the efficient killing of cancer in the clinic [3,4,5]. Mitochondria perform a variety of important cellular functions including the regulation of programmed cell death (i.e., apoptosis) via the caspase-9/caspase-3 pathway [6,7,8,9,10], and the production of the majority of cellular ATP needed for the endergonic processes of transmembrane drug efflux pumps in drug-resistant cancer cells [11,12]. Moreover, oxidative metabolism, which occurs in cancer cells [13], results in the production of a high amount of reactive oxygen species (ROS) from the mitochondrial electron transport chain (ETC). These high ROS levels activate signaling pathways that promote cancer cell proliferation and tumorigenesis, although cancer cells also produce high amounts of NADPH in the mitochondria to prevent a buildup of excessive ROS which can be toxic to cells [14,15,16,17,18,19,20,21,22]. In addition, mitochondria are the central regulators of cellular metabolism and the mediators of apoptosis [23]. Hence, mitochondria are crucial in mediating apoptotic cell death. Since cancer cells have suppressed apoptosis, mitochondria-directed delivery of drugs designed to trigger apoptosis are likely to be a promising strategy for treating cancer [24,25].

The era of chemotherapy began in the 1940s with the first uses of nitrogen mustards and antifolate drugs [26]. Although there has been a recent revolution of cancer drug development for targeted therapy and immunotherapy, chemotherapy is still a major therapeutic approach for cancer treatment in the clinic, which may be used alone or combined with other forms of therapy. Especially for cancers with no targeted therapy available such as triple-negative breast cancer and the majority of pancreatic cancer patients [27,28], standard chemotherapy is the current gold standard of treatment [29,30]. Although there might be more drug options available for cancer patients compared to targeted therapy or immunotherapy, the side effects of conventional chemotherapy of cancer are often severe due to its lack of specificity.

Recent studies show that engineered nanoparticles hold great promise to reduce side effects while improving the therapeutic efficacy of chemotherapy, particularly when it is combined with photodynamic and/or photothermal therapies. Various nanoparticle drug delivery systems (DDSs) have been developed in a broad range of clinical applications, and demonstrated tremendous potential for overcoming the limitations of free therapeutics [23,31,32,33,34,35]. Nanotechnology could help overcome several main limitations of conventional chemotherapy and photodynamic and/or photothermal therapies through tissue/cell-specific targeting, controlled release to specific subcellular organelles, and prolonged half-life in circulation. In view of the aforementioned critical role of mitochondria in controlling the fate of cells and the fact that there are prior comprehensive reviews on mitochondria-targeting materials/molecules [36,37,38] and mitochondrial biology [38,39], this mini-review surveys mitochondria-targeting nanoparticle drug delivery and its potential and challenges for advancing photodynamic, photothermal, and chemotherapies.

## 2. Fundamentals of Mitochondria-Targeting

Mitochondria are essential subcellular organelles for drug targeting due to their important roles in cell proliferation and death. They perform a variety of important cellular functions including the production of the majority of cellular ATP needed for endergonic processes, which enables the pumping action of the transmembrane drug efflux pumps in drug-resistant cancer cells [11,12]. They are also the central regulators of cellular metabolism and the mediators of apoptosis. Since mitochondria are crucial in mediating apoptotic cell death and cancer cells have suppressed apoptosis, mitochondria-directed delivery of drugs designed to trigger apoptosis are likely to be a promising strategy for treating cancer [24,25]. Therefore, targeting mitochondria has attracted great attention in medicine.

**Figure 1:**
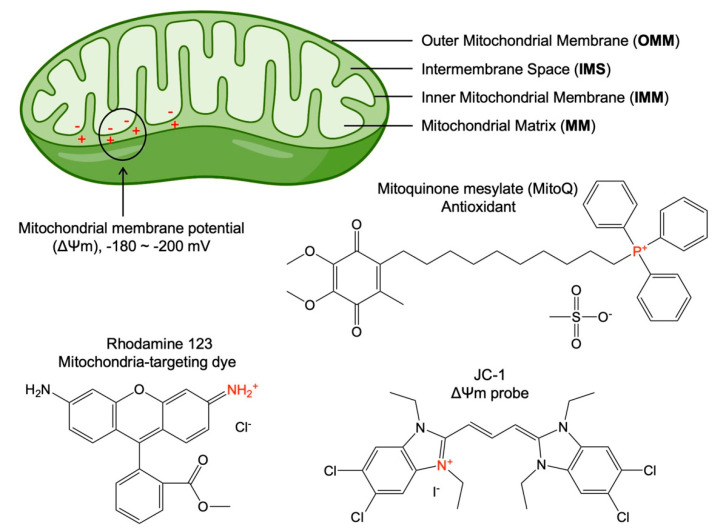
Schematic showing mitochondrial structure and three representative mitochondria-targeting molecules. The high mitochondrial membrane potential (∆Ψm) refers to the potential difference between the negative charges in the mitochondrial matrix (MM) and the positive charges in the intermembrane space (IMS), due to the proton pumps in the inner mitochondrial membrane (IMM) that pump protons from the MM into IMS. Also shown are three delocalized lipophilic cations, such as triphenylphosphonium (TPP)-based antioxidant mitoquinone mesylate (MitoQ) (a commercial mitochondria-targeting antioxidant), Rhodamine 123 (a mitochondria-specific fluorescent dye), and JC-1 (a mitochondrial membrane potential (∆Ψm) probe) that are the most well-established molecules that target mitochondria.

The idea of mitochondria-targeting was proposed in the 1950s after determining the mitochondrial structure and some molecules with high mitochondria affinity [40]. The structure of mitochondria is very different from other subcellular organelles in eukaryotic cells. This is because mitochondria contain four functional areas (Figure 1): the outer mitochondrial membrane (OMM), the intermembrane space (IMS), the inner mitochondrial membrane (IMM), and the mitochondrial matrix (MM) [41]. Due to the presence of proton pumps in the IMM, the protons in the MM are pumped into the IMS, resulting in positive charges in the IMS and negative charges in MM [41]. This difference in charges forms the transmembrane potential across the IMM, which is referred to as the mitochondrial membrane potential (∆Ψm, Figure 1). The potential difference on the two sides of the IMM is usually negative (approximately −180~−200 mV) [42]. Decades ago, a group of mitochondria-targeting small molecules were discovered for various biological applications by targeting the highly negative mitochondrial membrane potential. Although a few molecules (e.g., pyruvate [43] and glycyrrhetinic acid [44]) were reported for mitochondria-targeting, the most well-established mitochondria-targeting molecules were identified as delocalized lipophilic cations (Figure 1), such as triphenylphosphonium (TPP)-based antioxidant mitoquinone mesylate (MitoQ), 5,5′,6,6′-tetrachloro-1,1′,3,3′-tetraethyl-imidacarbocyanine (JC-1), and rhodamine 123 [45,46,47]. The lipid solubility of these molecules enables them to cross the cell membrane and mitochondrial membrane, and the positive charge enables them to enter MM under the action of the mitochondrial membrane potential, rendering them with mitochondria-targeting and accumulating ability.

The most successful case of using lipophilic cations for mitochondria-targeting is the discovery of TPP [45]. TPP has a positively charged phosphorus atom (i.e., is cationic) surrounded by three hydrophobic phenyl groups that render it lipophilic [48]. TPP has been investigated as a mitochondria-targeting probe for over 40 years. Due to the highly negatively charged mitochondria membrane potential of about −180 mV, there is a preferential accumulation of TPP into the mitochondrial compartment space [45]. TPP-based mitochondria-targeting is advantageous compared to other approaches for mitochondria-targeting; because it is stable in biological systems, relatively simple to synthesize and purify, low in chemical reactivity with cellular components, and with minimal light absorption or fluorescence in the near-infrared (NIR) spectral region [49]. TPP targets and penetrates the mitochondrial membranes in a multi-step process: it binds to the outer mitochondrial membrane (OMM), then moves to and crosses the inner mitochondrial membrane (IMM), and finally dissociates from IMM. The cationic structure naturally allows for electrostatic targeting and accumulation inside the mitochondrial matrix which has a highly negative membrane potential, compared to the cell membrane potential (−25 mV) [47]. Due to the highly specific and efficient mitochondria-targeting properties of lipophilic cations, such as TPP, the biological applications of such a simple and effective strategy for mitochondria-specific delivery are unlimited. The mitochondria-targeting ability of TPP has enabled the engineering of successful cancer therapies designed to eradicate cancer cells through mitochondria-dependent apoptosis [45,50].

## 3. Mitochondria-Targeting Nanoparticles for Advancing Cancer Therapies

### 3.1. Mitochondria-Targeting in Photodynamic Therapy for Cancer

**Figure 2:**
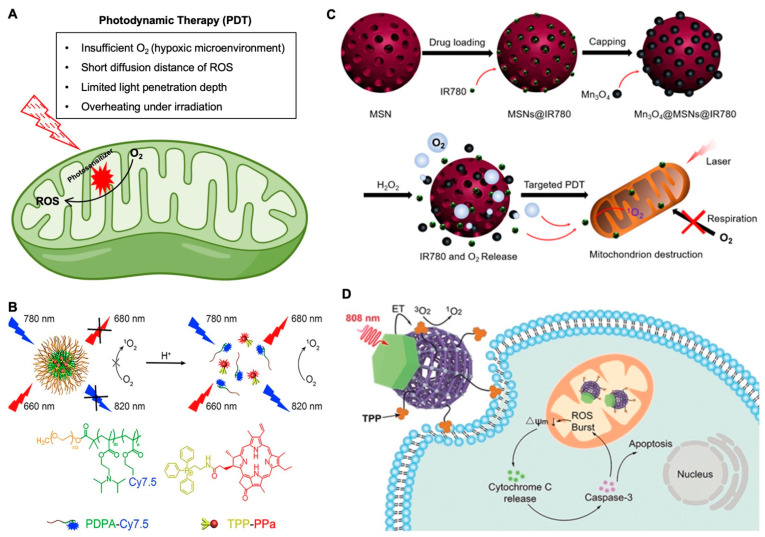
Mitochondria-targeting in photodynamic therapy. (**A**) A schematic illustration of mitochondria-targeting photodynamic therapy and its major challenges. (**B**) A pH-activatable mesoporous silica nanoparticle (MSN)-based platform for dual-stage precisely mitochondria-targeted photodynamic anticancer therapy [60]. (**C**) Self-generating oxygen enhanced mitochondria-targeted photodynamic therapy for tumor treatment with hypoxia scavenging [61]. (**D**) Nd3+-Sensitized upconversion metal-organic frameworks for mitochondria-targeted amplified photodynamic therapy [69]. Reproduced with permission from references [60] (Copyright 2019 by Elsevier), [61] (Open access article distributed under the terms of the Creative Commons Attribution License), and [69] (Copyright 2019 by John Wiley and Sons) for the images in (**B**–**D**), respectively.

Photodynamic therapy (PDT) is based on the generation of ROS by photosensitizers (PSs) under the irradiation of light to destroy cancer cells. Unfortunately, as summarized in Figure 2A, the therapeutic effect of PDT is limited by both the short (<20 nm) diffusion distance of ROS from the PSs and the insufficient oxygen in the hypoxic tumor microenvironment [51,52,53,54,55], and the short penetration depth of the light being used for PDT also significantly affects its therapeutic efficacy [56]. In one study, dendrimer-based upconversion nanoparticles (39.5 nm in diameter and 13.6 mV in surface zeta potential) were used to enhance penetration depth, and stabilize the nanoparticles against photobleaching [57]. In addition, to overcome the shortage of O_2_ that is needed for type II PDT in the hypoxic tumor microenvironment, catalase was incorporated into the nanoparticles to promote the conversion of H_2_O_2_ into O_2_ to enhance the therapeutic efficacy of PDT for destroying tumors. Finally, TPP was used to target the mitochondria since intracellular ROS could cause cell necrosis by damaging the mitochondria and DNA. In vitro studies showed that the mitochondria-targeting nanoparticles were able to induce apoptosis more effectively than the non-mitochondria-targeting nanoparticles. This suggests that the mitochondria-targeting capability enhances the ROS generation-based cell killing in PDT. In their in vivo study, the greatest tumor inhibition was observed in the treatment group with mitochondria-targeting ability, suggesting that mitochondria-targeting can enhance PDT even in vivo.

Another tool that can be used efficaciously in mitochondria-targeting is 2D nanomaterial sheets. The direct use of 2D metal-free semiconductor nanomaterials is limited by the inefficient photocatalytic activity and visible light-responsive properties [58]. A 2D heterostructure with TPP modification (~200 nm in diameter and 7.6 mV in surface zeta potential) was used to act as type I PDT (oxygen-independent) in addition to a type II PDT (oxygen-dependent) via the splitting of endogenous water to self-supplement O_2_ for type II PDT [59]. Using JC-1 staining to determine the state of the mitochondria membrane potential, the data showed that the nanosheets with mitochondria-targeting capability accumulated in the mitochondria more efficiently and induced more mitochondrial damage than non-mitochondria-targeting nanosheets. In the in vivo study, only the treatment with mitochondria-targeting nanosheets completely eradicated the entire tumor without any recurrence for two weeks. Moreover, the mitochondria-targeting nanosheets led to the highest levels of cell necrosis in the tumors. This suggests the strong benefits of applying mitochondria-targeting to enhance PDT efficacy.

To overcome the poor endosomal escape and delivery efficacy of many nanoparticle systems, a pH-activable nanoparticle (~24.33 nm in diameter and approximately −9 mV in surface zeta potential) was designed for efficient endosomal escape and targeting of the mitochondria for fluorescence imaging and enhanced PDT efficacy [60], as shown in Figure 2B. In the work, a copolymer was labeled with fluorescent dyes and a mitochondria-targeted PS. The nanoparticle was designed to undergo a rapid destabilization in the endosomes due to the ultra pH sensitivity of the polymer followed by the release of the TPP-pyropheophorbride-a (TPPa) into the cytosol, targeting and entering the mitochondria, ROS species production (upon laser irradiation) in mitochondria, dysfunction of mitochondria, and cancer cell apoptosis. The data demonstrates that the mitochondria-targeting effect enhances the efficacy of the treatment. In the in vivo mouse study, the mitochondria-targeting nanoparticle with TPP conjugation resulted in better tumor inhibition than the nanoparticle without mitochondria-targeting capability. Moreover, the nanoparticle with mitochondria-targeting induced the lowest level of cancer-associated fibroblasts, the lowest level of Ki67-positive proliferating cells, and the highest level of TUNEL-positive apoptotic cells in the ovarian tumor tissues. This work demonstrated a highly pH-sensitive nanoparticle platform that can treat tumors efficiently with endosomal-escaping and mitochondrial-targeting properties.

Another study, as shown in Figure 2C, focused on overcoming tumor hypoxia as a limitation of PDT, developed mesoporous silica nanoparticles (90 nm in diameter and 3 mV in surface zeta potential) encapsulated with a PS (IR780) and capped with Mn_3_O_4_ nanoparticles (5 nm in diameter and 35 mV in surface zeta potential) [61]. These nanoparticles were intelligently designed to decompose the H_2_O_2_ in tumor cells into O_2_ via Mn_3_O_4_. After the decomposition of the Mn_3_O_4_ nanoparticles with the conversion of H_2_O_2_ into O_2_, the IR780 can be released into the cytosol to efficiently target the mitochondria via the intrinsic lipophilic cationic properties of the molecule. Afterwards, 808 nm laser irradiation was used to generate ROS in the mitochondria, cause their dysfunction, and significantly induce apoptosis of the cell. This work demonstrated that targeting of the mitochondria leads to mitochondria respiratory inhibition, which can reverse the hypoxia in the tumor, in contrast to conventional PDT that consumes oxygen in the tumor microenvironment to exacerbate the hypoxic environment in a tumor. Both the in vitro and in vivo data suggested that the nanoparticles can efficiently kill cancer cells and ameliorate the hypoxic tumor microenvironment. The nanoparticles with laser treatment resulted in the highest levels of oxygenated hemoglobin and lowest levels of non-oxygenated hemoglobin in the tumor tissue 24 h after intravenous (IV) injection. Moreover, there was no detectable increase in the HIF-1α levels of the nanoparticles with laser treatment compared to the control groups, which suggests that the treatment inhibits the hypoxia signaling pathway of the cancer cells. Moreover, the nanoparticle treatment with laser irradiation led to a significantly increased inhibition of tumor growth, compared to the other treatments, suggesting that the mitochondria-targeting and suppression of the tumor hypoxia augment tumor killing.

One major challenge to PDT can be an overheating effect from the laser. This happens with many upconversion nanoparticle-based PDT systems as a result of their superior heating effect [62]. Many upconversion nanoparticle-based PDTs are excited using a 980 nm laser to provide good tissue penetration. However, water has a noticeable absorption of light at and beyond 980 nm which can result in tissue overheating during the PDT treatment. This overheating issue caused by the laser can lead to non-specific killing of healthy cells that may be adjacent to cancer cells, which can be harmful to the patient and overall limit the therapeutic benefit of the PDT. As mentioned previously, upconversion nanoparticles are becoming increasingly popular as nanoparticles that can upconvert two or more low-energy photons into one high-energy photon [62,63,64,65,66,67]. Mitochondria-targeting could also amplify the PDT efficacy through the depolarization of the mitochondria membrane and the initiation of the intrinsic apoptotic pathway [68]. Using metal-organic frameworks (MOFs) and upconversion photochemistry with mitochondria-targeting, Janus nanostructures (~76 nm in diameter) were developed with asymmetric functionalization, which could allow for photosensitizing with 808 nm NIR light and avoid the laser-irradiation overheating effect (Figure 2D) [69]. These nanoparticles feature Nd^3+^-sensitive upconversion of the MOFs with TPP-surface functionalization for mitochondria-targeting. Their imaging studies with confocal microscopy highlighted that there is increased overlap between the mitochondria and nanoparticles with the TPP modification, which validates that the MOFs are being driven into the mitochondria. Additionally, much enhanced killing of the cancer cells was observed for the mitochondria-targeted MOFs. Cytochrome C and caspase-3, two proteins in the apoptotic cascade, showed much higher expression in the cells treated with the mitochondria-targeted MOFs than in the cells treated with MOFs without any mitochondria-targeting capability. In the in vivo study with mice bearing 4T1 tumors, the mitochondria-targeted MOF treatment resulted in the greatest inhibition of tumor growth during the 14-day period of observation, suggesting that mitochondria-targeting can greatly enhance the PDT efficacy. The authors emphasized that they were able to overcome the overheating problem of many upconversion nanoparticle-based PDT systems quite robustly. Compared with the 980 nm laser that increased the temperature of the skin by 6.8 °C after irradiation for 5 min, the 808 nm laser did not cause obvious change in skin temperature even after 10 min of irradiation given other conditions being the same. Overall, this work demonstrated a sophisticated PDT that can overcome major barriers to PDT using a shifted irradiation wavelength and mitochondria-targeting.

To overcome the aggregation-induced quenching phenomenon that reduces ROS generation of fluorescent photosensitizers and the efficiency of PDT, the development of fluorescent photosensitizers with AIE (aggregation-induced emission) characteristics has led to much interest because they show highly efficient ROS production in the aggregated state [70,71,72]. Zheng et al. generated new AIE cross-linked copolymer-based nanoparticles (260 nm in diameter and 39 mV in surface zeta potential) with far-red (FR)/NIR bioimaging capability and efficient ROS generation for mitochondria-targeted and image guided-PDT [73]. These nanoparticles were able to generate ROS upon a general LED white light irradiation with an excellent ROS quantum yield of 77.9%, which is much higher than that of clinically used photosensitizers Photofrin (28%) and Laserphyrin (48%). The nanoparticle with TPP modification was shown to have significantly higher mitochondria-targeting ability and cancer cell killing effect than that without TPP. This highly effective platform provides a new approach for mitochondria-targeted PDT with an inexpensive ultralow-power white light irradiation.

### 3.2. Mitochondria-Targeting in Photothermal and Magnetothermal Therapies of Cancer

**Figure 3:**
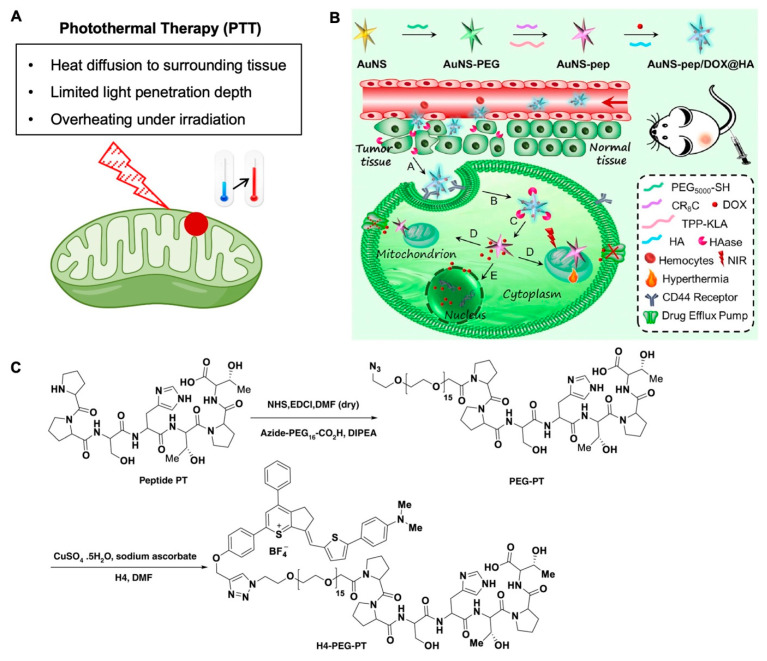
Mitochondria-targeting in photothermal therapy. (**A**) A schematic illustration of mitochondria-targeting photothermal therapy and its major challenges. (**B**) A mitochondria-targeting “Nanoheater”, gold nanostar (AuNS), for enhanced photothermal/chemotherapy [85]. (**C**) Synthesis route of an upconversion NIR-II fluorophore for mitochondria-targeted cancer imaging and photothermal therapy [86]. Reproduced with permission from references [85] (Copyright 2019 by Elsevier) and [86] (Open access article distributed under the terms of the Creative Commons Attribution License) for the images in (**B**,**C**), respectively.

Photothermal therapy (PTT) kills cancer cells/tumors by converting the electromagnetic energy of light into thermal energy via a photosensitizer to heat the cells/tumors (Figure 3A). Although it is designed to generate heat only in the targeted areas with a photosensitizer, PTT may also non-specifically kill non-targeted surrounding tissues due to heat diffusion or unintended delivery of photosensitizer into the surrounding tissue. The mitigation of this shortcoming in PTT has been investigated widely [74,75,76,77,78,79,80,81,82]. One method that has shown some promise in avoiding the detrimental side effects of PTT is to use mitochondria-targeting nanomaterials [50].

Nanomaterials for phototherapy can be divided into three types: organic, inorganic, and hybridized with both organic and inorganic components [83,84]. In a study that highlights the benefits of using mitochondria-targeting PTT for cancer therapy (Figure 3B) [85], gold nanostars (AuNSs) were co-encapsulated with a widely used chemotherapy drug, doxorubicin (DOX), inside a hyaluronic acid (HA) shell for tumor-targeting photothermal chemotherapy (94.6 nm in diameter and −13.1 mV in surface zeta potential). The AuNSs were used to enhance the local surface plasmon for efficient photothermal conversion under NIR irradiation. TPP modified with a pro-apoptotic peptide was applied to target mitochondria and induce mitochondria-dependent apoptosis of the cell. It was demonstrated that the mitochondria-targeting and pro-apoptotic peptide enhance cell killing compared to a non-mitochondria-targeting treatment. Moreover, there was slight toxicity of the nanoplatform with the mitochondria-targeting and pro-apoptotic peptide modification to the AuNS when no light was irradiated. Significantly, the DOX retention in the cells for the nanoplatform with TPP and peptide-modified AuNS was ~89% of the initial dose compared to free DOX treatment which only retained 45% of the initial DOX dosage. Additionally, mitochondria-targeting was confirmed by the presence of star-shaped structures in the mitochondria, and JC-1 staining indicated a low mitochondria transmembrane potential after treatment with the nanoplatform. This work suggests the mitochondria-targeting combined with pro-apoptotic peptide can enhance the therapeutic effect of DOX-mediated chemotherapy and gold nanoparticle-mediated photothermal therapy.

Recently, a group [86] used an emerging class of fluorophores that emits light in the second NIR window (NIR-II, 1000–1700 nm) to enhance the penetration of the light in biological tissue [87,88,89,90,91]. In this work, these NIR-II fluorophores were engineered to have a mitochondria-targeting capability and overcome several disadvantages of other mitochondria-targeting fluorescence dyes (Figure 3C) [86], which mostly emit only one color of light in the visible or NIR wavelength [92,93,94,95,96,97,98,99,100,101,102,103]. Moreover, these dyes mostly have poor photostability or small Stokes shifts. The fluorophore was used to stain mitochondria for imaging (peak emission wavelength of ~1100 nm), in addition to having excellent photothermal efficiency, and very deep tissue NIR-II imaging of osteosarcoma in vivo. The results indicated that the fluorophore was able to target the mitochondria and induce mitochondria dysfunction when irradiated with an 808 nm laser to emit light in the NIR-II window. Moreover, the fluorophore was able to efficiently and robustly shrink tumors in an osteosarcoma mouse model only when both the fluorophore and laser were applied together. Overall, this work demonstrated the benefits of mitochondria-targeting in an antitumor photothermal therapy approach.

Additionally, both PTT and photodynamic therapy (PDT) can be used concurrently to elicit an antitumor effect [104]. Black phosphorous (BP) has become a favorable inorganic material for phototherapy due to its biocompatibility and efficient ROS generation as a PS [105,106]. One group used BP-based nanomaterials to provide both a photothermal and photodynamic therapeutic-based approach for cancer therapy [107]. They conjugated the nanoparticles with polydopamine for photothermal conversion and enhanced stability of the BP nanosheets (213.9 nm in diameter). Polydopamine has been investigated as a superior polymer for photothermal applications since it has been shown to have excellent free radical scavenging [108] and exhibits a photothermal energy conversion efficiency of ~40%, which is much higher than that (~22%) of conventionally used gold nanorods [109]. Since BP has very high absorption in the NIR spectrum, it allows for a very efficient photothermal property as well. TPP was covalently conjugated to the surface of the nanosheets for mitochondria-targeting. The nanosheets were incubated with HeLa cells for them to take up the nanomaterial, irradiated with laser, and then further incubated before measuring viability. It was found that the BP nanosheets conjugated with TPP could kill the cancer cells much better than BP nanosheets without any TPP, indicating the mitochondria-targeting enhances the photothermal effect of the nanomaterials. For in vivo studies, the nanosheets with TPP conjugation completely ablated the tumor, which was attributed to the mitochondria-targeting ability. Without TPP conjugation, the tumor was not completely destroyed, but still vastly shrunk. Toluidine blue (TB) is a biocompatible and cationic photosensitizer that can accumulate in the negatively charged mitochondria after entering cells. TB is not only a photothermal agent but also a safe and effective photosensitizer. Yang et al. designed and synthesized TB nanoparticles (232.6 nm in diameter and approximately −18 mV in surface zeta potential) with uniform size, good stability, and excellent therapeutic effect [110]. In an in vivo study, the tumor temperature was recorded, and the temperature of the nanoparticle treated sample was shown to be significantly higher than that of the control and TB only groups under the same light conditions. After 5 min of illumination (660 nm laser), the temperature increased by ~22 °C. The results show that TB nanoparticles accumulate in the tumor effectively and produce a heat-induced killing effect. In their tumor ablation study, the combination of the nanoparticle and laser treatments could effectively destroy the tumor, and in two of the mice (out of 5), the tumors were completely ablated. Additionally, the light irradiated TB group showed distinct cell necrosis, in addition to unclear cell boundaries, loose tumor tissue arrangement, cell rupture, and increased mitotic phase. The necrosis of the tumor cells was more evident in the NPs group with laser irradiation than in other groups.

Mitochondria-targeting has also been applied to magnetothermogenic nanomaterials to elicit an augmented therapeutic effect [111,112]. A magnetic field can penetrate deep into all tissues, thus overcoming a major weakness of photothermal therapy. Deep tumors may be treated with magnetothermal therapy without significant damage to surrounding healthy tissues [113,114]. Shen et al. used an iridium (III) complex as a mitochondria-targeting agent on the surface of MnFe_2_O_4_ nanoparticles (~11 nm in diameter and ~19 mV in surface zeta potential) for a magnetic hyperthermia treatment (MHT) and chemodynamic therapy against cancer [115]. After exposure to a magnetic field, the magnetothermogenic nanoparticls induced a localized increase in temperature to cause mitochondrial damage. Glutathione (GSH) reduces Fe(III) to Fe(II) on the nanoparticle surface, which additionally catalyzes the conversion of H_2_O_2_ into cytotoxic OH free radicals via the Fenton reaction. This in situ generation of OH helps to overcome the limited penetration depth of PDT. Moreover, the iridium (III) complexes have excellent two-photon absorption and are superior to one-photon imaging in terms of red-shifted absorption, enhanced tissue penetration, reduced photobleaching, prolonged observation time, and improved spatial resolution. These results suggest that the nanoparticles have excellent magnetothermal properties and can be used to achieve non-invasive and highly localized therapy. The magnetothermogenic nanozyme nanoparticles were injected in vivo into mice bearing xenografted HeLa tumors. The nanoparticles with an alternating magnetic field are the only treatment to cause a decrease in tumor volume. A statistically significant increased expression of HSP70 was also observed for this treatment compared to the other control treatments. These results demonstrate that the nanoparticles increase tumor temperature with the alternating magnetic field, leading to the increased HSP70 expression. Magnetothermogenic nanomaterials are a tremendous tool that can overcome several barriers of photothermal therapies and can be modified for mitochondria-targeting for localized heating and increased killing of cancer cells.

### 3.3. Mitochondria-Targeting in Chemotherapy

Due to the success of mitochondria-targeting in chemotherapy applications, there has been exploration into some novel targeting ligands that can also boost mitochondrial permeability and enhance the cargo uptake into mitochondria [43,45,116,117,118,119,120,121,122]. Glycyrrhetinic acid (GA), a natural product from licorice, was found to target mitochondria [44]. Moreover, it has been reported that GA interacts with the mitochondrial respiratory chain, resulting in the generation of hydrogen peroxide. The hydrogen peroxide oxidizes the thiol groups and endogenous pyridine nucleotides, leading to the opening of mitochondria permeability transition pores [123,124]. GA was functionalized onto graphene oxide (GO) and used as a nanocarrier to deliver DOX into the mitochondria of cancer cells and overcome some barriers to mitochondria delivery such as low permeability of the mitochondrial membrane (200 nm in diameter and approximately −37.6 mV in surface zeta potential). Moreover, GA is reported to exhibit antitumor activity as well [125]. It was demonstrated that there was a highly targeted delivery of DOX to the mitochondria of HepG2 cells with the GA functionalized GO, and without the mitochondria-targeting characteristic of the nanoparticles, there was nearly no DOX localization to the mitochondria. Furthermore, the ratio of Bax (pro-apoptotic protein) to Bcl-2 (anti-apoptotic protein) was investigated as these are two important regulators of mitochondria-mediated caspase activation. The ratio of Bax/Bcl-2 for the GA functionalized nanoparticles was 10.5-fold greater than that of the control group with no treatment, whereas the non-GA functionalized nanoparticles had a 4.1-fold greater ratio than the control group. Additionally, the in vivo results showed that the mitochondria-targeting benefit was evident in enhancing the chemotherapeutic potential of this nanocarrier.

Interestingly, Xu et al. designed and synthesized several dual-targeting (both cellular and subcellular targets) DDSs, aiming to enhance the accumulation of anticancer agents into mitochondria of cancer cells to amplify their anticancer effect [24,50,126]. For example, a polypyrrole-silica (Py@Si)-based hybrid nanoparticle (~75 nm in diameter and approximately −13.3 mV in surface zeta potential) was developed for targeting not only CD44-overexpressed cancer cells but also their mitochondria [50]. The nanoparticle enables precise control of DOX release in tumors and generates heat within mitochondria under 808 nm laser irradiation. Unlike other phototherapy nanosystems, this strategy uses only a mild photothermal effect to trigger DOX release and heat up mitochondria. Both in vitro and in vivo data indicated that targeted damage of mitochondria greatly sensitizes cancer cells to chemotherapy, suggesting a promising strategy for combating cancer.

Superparamagnetic iron oxide nanoparticles (SPIONs) are highly desired for use in the human body compared with other metal oxide nanoparticles due to their high durability, biocompatibility, biodegradability, and low toxicity. The low toxicity of SPIONs to healthy/normal cells offers a critical advantage, compared to other inorganic nanoparticles. SPIONs are also able to cross the nuclear membrane [127] and generate ROS [128,129]. In a study by Afrasiabi et al. [130], SPIONs were shown to generate more ROS in oral squamous cell carcinoma (OSCC) mitochondria than in normal cell mitochondria. This is attributed to cancer cells being more sensitive to free radicals and oxidative stress than normal cells. Additionally, the concentration of cytochrome C was significantly greater in OSCC mitochondria treated with SPIONs than healthy cell mitochondria treated with SPIONs. Cytochrome C release is one of the earliest, irreversible events in mitochondria-mediated apoptosis signaling, suggesting that the SPIONs can be used for mitochondria-targeting in cancer cells. SPIONs induce much higher cytotoxicity against OSCC cells than normal, healthy cells, and the toxicity of SPIONs to normal, healthy cells is negligible, except for the highest dosage of SPIONs (400 nM). In addition, SPIONs increased caspase-3 activity in OSCC cells significantly while non-significant change in the caspase-3 activity was observed in SPIONs-treated normal, healthy cells. This work demonstrates that SPION treatment is associated with an increase in ROS levels, decrease in viability, and increase in caspase-3 activity in OSCC cells. Therefore, SPIONs hold potential as a mitochondria-targeting platform for cancer therapies.

Moreover, besides the aforementioned inorganic materials (excluding GA), organic materials such as polymers and lipids have been used in mitochondria-targeted nanotechnologies. A TPP-modified polymer was used in the work of Wang et al. in which PF127 (poly(ethylene oxide)-block-poly(propylene oxide)-block-poly(ethylene oxide), PEO-PPO-PEO triblock copolymer) was modified with TPP and HA to neutralize the positive charges of the TPP in order to avoid rapid clearance and achieve a long-term circulation [131]. The HA was grafted to the OH-PF127-TPP via covalent bonding with the OH group. The resultant block copolymer was then used to make nanomicelles (142 nm in diameter and −24.65 mV in surface zeta potential) with a chemotherapeutic drug, paclitaxel (PTX), being loaded in the hydrophobic core of the nanomicelles to deliver a toxic reagent to the mitochondria of cancer cells. These nanomicelles were used to target tumor cells via the HA affinity towards the CD44 receptors overexpressed on cancer cells. In addition, the nanomicelles can target mitochondria efficiently via TPP and thus offer dual-targeting of both the cancer cells and mitochondria. After targeting the CD44 receptors on cancer cells, the HA on the outer shell of the nanomicelles is degraded via hyaluronidase inside the cancer cells and then after endosomal escape, the nanomicelles can efficiently localize in the mitochondria of the cancer cells after 24 h of incubation. The delivery of PTX to the mitochondria also caused the permeabilization of the mitochondrial outer membrane via the inhibition of Bcl-2 signaling, leading to cytochrome C release and activation of caspase-3 and caspase-9. In a xenograft breast cancer-bearing mouse model of lung metastasis, the nanomicelles with both TPP- and HA-modified PF127 showed the most obvious tumor-targeting and significantly improved antitumor efficacy, compared to the micelles without HA.

In Kuznetsova et al. [132], cationic liposomes of alkyltriphenylphosphonium bromides with different hydrocarbon tail lengths were used as mitochondria-targeting agents for targeted delivery to mitochondria. It was found that increasing the alkyl tail length of the alkyltriphenylphosphonium bromide leads to an increase in the positive charge of the liposomes. The liposomes (100 nm in diameter and 35–46 mV in surface zeta potential) were used to encapsulate hydrophilic agents including metronidazole and DOX. The toxicity of the liposomes to cancer cells was analyzed and it was shown that the 1,2-dipalmitoyl-sn-glycero-3 phosphocholine liposomes synthesized in this work performed better than the traditionally used dipalmitoylphosphatidylcholine (DPPC) liposomes. More specifically, DOX encapsulated in the TPPB-14/DPPC liposomes exhibited an IC50 (inhibitory concentration for killing 50% of cancer cells) that was approximately half of the drug encapsulated in the conventional DPPC liposomes.

## 4. Outlook and Conclusions

Mitochondria have critical roles in tumor progression because they not only produce ATP to supply the energy needed for rapidly proliferating cancer cells, but also provide the complex molecules for anabolism, their capacity to produce ROS, their resistance to adverse microenvironmental conditions, interaction with tumor stroma, and metastatic potential, which all rely on optimal mitochondrial biogenesis and oxidative phosphorylation [133]. Mitochondria-targeting can greatly benefit multiple types of DDSs as cited in the preceding sections. Several kinds of nanomaterials including polymeric, lipid, semiconductor, magnetic, silica, graphene oxide, and metallic nanoparticles have been used for mitochondria-targeted cancer therapies to enhance cancer cell destruction. With new mitochondria-targeting nanocarriers being developed for the delivery of PDT, PTT, and traditional chemotherapy agents [134,135], this is a strategy that should be applied to new cancer therapies to efficiently augment mitochondria-mediated apoptosis. As PTT and PDT methodologies become more precise, safe (targets tumor and avoids overheating of healthy tissue), and tailored to the tumor microenvironment (hypoxia and low pH), mitochondria-targeting will be an important function to implement in the delivery of these ablation agents for an augmented therapeutic effect that may be translatable for clinical application. Furthermore, as the fundamental understanding of mitochondria-enabled tumorigenic and cancer cell survival mechanisms is improved, it may be possible in the near future to target these mechanisms inside specific compartments of the mitochondria and elicit a more powerful antitumor effect. In addition, furthering our understanding of the mitochondria structure and function can allow for a more diverse set of mitochondria-targeting ligands to be developed with even greater specificity. Mitochondrial-targeting biomolecules [136,137,138,139,140,141,142], such as some peptides, show promise as mitochondria-targeting molecules with no detrimental effects on cells; however, the targeting mechanism of these molecules is unclear and should be elucidated further. With many diseases being linked to mitochondrial dysfunction [143], including neurodegenerative diseases, it is imperative to expedite the development of mitochondrial-targeting nanomaterials for drug delivery to improve the efficacy and safety of treating these diseases.
